# Psychiatric Symptomatology, Mood Regulation, and Resting State Functional Connectivity of the Amygdala: Preliminary Findings in Youth With Mood Disorders and Childhood Trauma

**DOI:** 10.3389/fpsyt.2020.525064

**Published:** 2020-09-18

**Authors:** Yael Dvir, David N. Kennedy, Steven M. Hodge, Destiny Pegram, Brian Denietolis, Jean A. Frazier

**Affiliations:** ^1^ Psychiatry/Child and Adolescent Psychiatry, University of Massachusetts Medical School, Worcester, MA, United States; ^2^ Eunice Kennedy Shriver Center, University of Massachusetts Medical School, Worcester, MA, United States

**Keywords:** mood regulation, resting state functional connectivity, amygdala, trauma, magnetic resonance imaging

## Abstract

**Background:**

As mood dysregulation and hyperarousal are overlapping and prominent features of posttraumatic stress disorder (PTSD), and mood disorders (MD) including bipolar disorder (BD), we aimed to clarify the role of trauma and MD on the resting state functional connectivity (RSFC) of amygdala in MD youth with or without trauma exposure, and healthy controls (HC).

**Methods:**

Of 23 subjects, 21 completed the magnetic resonance imaging (MRI) protocol, 5 were excluded for subject motion, leaving final sample size of 16: nine subjects with MD (5/9 with trauma), and 7 HC. Youth were assessed with Schedule for Affective Disorders and Schizophrenia for School Aged Children—Present and Lifetime Version (K-SADS-PL), and other behavioral measures including Young Mania Rating Scale (YMRS). Imaging data were acquired using functional MRI in 3-T scanner. Imaging included T1-weighted structural MRI and 6-min resting state acquisition.

**Results:**

In between group analysis, the average correlation coefficients between left anterior cingulate cortex (Acc) and left insula cortex with left amygdala regions were significantly larger in HC compared to the patient population. Connectivity between left amygdala and left cingulate cortex shows a significant negative correlation with YMRS severity.

**Conclusions:**

In this preliminary study, MD with trauma youth had more manic symptoms and difficulties regulating anger. While MD youth showed reduced RSFC of left amygdala with left acc and left insula, no significant difference between the subgroups of children with MD was observed. However, when looking at both clinical groups together, we observed a significant correlation of RSFC of left amygdala to left acc, and YMRS scores.

## Introduction

Identifying the contributions of childhood trauma to the development and presentation of mood disorders (MD) is an important task for clinicians working with affected youth (1), especially given known contributions of childhood trauma to mood dysregulation and more severe presentations of MD (2). Assessing for biomarkers of illness by using neuroimaging is a powerful way to address this diagnostic quandary. Given that deficits in emotion processing and hyperarousal symptoms are overlapping and prominent features of posttraumatic stress disorder (PTSD), and MD including bipolar disorder (BD), studies are needed in order to highlight disorder-unique versus common psychopathologies. We sought to clarify the role of trauma and MD on the resting state functional connectivity (RSFC) of the amygdala in youth with MD with or without trauma exposure as well as healthy controls (HC).

Understanding the association between trauma and the development of MD will increase our knowledge of the diverse effects of such events on youths’ emotional and behavioral development. Specifically, it is important to look at mood dysregulation as a central symptom across diagnostic groups, as mood regulation/dysregulation may have important implications in terms of treatment approaches, biological markers, and social/demographic factors. In this context, we are specifically interested in investigating the brain function of the amygdala, a brain region which mediates aspects of social-emotional functioning, and participates in processing information about significant emotional stimuli.

### Amygdala and RSFC in Youth With MD

Relative to HC, several RSFC studies have shown that youth diagnosed with major depressive disorder (MDD) exhibit hypoconnectivity between the amygdala and the dorsolateral prefrontal cortex and the anterior insula (3). Bebko and colleagues (4) showed a trend toward an inverse relationship between the RSFC between the amygdala-bilateral posterior insula and the parent general behavior inventory item scale, demonstrating that youth who exhibited increasingly dysregulated behaviors had lower functional connectivity between the amygdala-bilateral insula (4). Greater positive connectivity between the ipsilateral amygdala and insula has been associated with greater remission in depressive symptoms over time (5). When comparing the functional connectivity of patients with MDD who completed cognitive behavioral therapy (CBT) treatment, these individuals display increased connectivity of their amygdala to their insula, anterior cingulate cortex (Acc), and dorsolateral prefrontal cortex relative to their pre-CBT connectivities (3). Finally, in a study by Pannekoek and colleagues (6), the left amygdala demonstrated hyperconnectivity to the inferior frontal gyrus in individuals with depression (6).

### Amygdala Connectivity in Traumatized Adolescents

Thomason and colleagues (7) showed that there was a decrease in negative connectivity between the insula and the amygdala in urban youth who had experienced childhood maltreatment, suggesting reduced emotion regulation control/loss of inhibitory affective control in those youth (7). Marusak and colleagues (8) demonstrated that trauma exposed youth had increased connectivity within the amygdala and insula relative to youth not exposed to trauma (8). Reduction in amygdala-insula connectivity during tasks that require cognitive reappraisal have been correlated with greater symptom reduction in adolescent girls treated with Trauma Focused CBT. However, girls that continued to exhibit increased amygdala-insula connectivity during tasks that require cognitive reappraisal had less abatement of symptoms associated with PTSD. The authors hypothesize that this change indicates decreased interoceptive representation of negative affective states (9).

Adolescents with PTSD have been shown to exhibit hypo-connectivity between amygdala and frontal structures including the dorsolateral and ventromedial cortex, Acc, and hippocampus. Herringa and colleagues (10) found Childhood Trauma Questionnaire (CTQ) scores to be inversely correlated with the RSFC of right amygdala and subgenual Acc. This study also showed that reduced amygdala-hippocampal connectivity was associated with increased internalizing symptoms (10).

### Amygdala Connectivity in MD and Trauma

Few studies have researched RSFC in co-occurring PTSD and MD in adolescents. Sun and colleagues (11) examined the effects of early life abuse as measured by CTQ in adolescents with depression who were overweight. The study demonstrated decreased connection between the amygdala and precuneus, with less negative connectivity in adolescents with depression who were overweight and experienced high levels of abuse relative to adolescents with depression who were overweight and experienced low levels of abuse. Those with depression who were overweight and experienced high levels of abuse exhibited decreased connectivity, and less negative interaction between the insula and the precuneus relative to their low-level abuse counterparts (11).

In adults with co-occurring PTSD and MDD, Kennis and colleagues (12) demonstrated increased RSFC between the hippocampus and the insula relative to subjects with PTSD alone. Of note, the difference between the two groups became insignificant when subjects taking psychotropics were excluded from this analysis. This study also showed increased connectivity between the subgenual Acc and the perigenual Acc in the PTSD+MDD group. There was a negative correlation between the connectivity of these two structures and re-experiencing symptoms of PTSD (12). Zhu and colleagues (13) also sought to study differences in RSFC in adults with co-occurring PTSD and MDD, PTSD alone, and trauma-exposed healthy individuals. In this study, individuals with PTSD+MDD versus PTSD alone exhibited significantly decreased RSFC in basolateral amygdala and orbitofrontal cortex. This connectivity inversely correlated to severity of MDD symptoms in all three subject groups (13). Satterthwaite and colleagues (14) examined the relationship between depressive symptoms in women with the diagnosis of MDD, PTSD, and HC and resting connectivity and showed depression severity to be linked to decreased RSFC between amygdala and anterior insula, dorsolateral prefrontal cortex, and Acc (14).

### Hypothesis

Given this literature and our prior expectations, we hypothesize that children with MD trauma histories will exhibit abnormal connectivity between amygdala and frontal lobe, when compared to children with MD without trauma and HC.

## Approach

### Subjects

We studied three samples consisting of 8- to 12 year-old children: 1) MDT: Children who have experienced trauma (parent reported history of significant interpersonal trauma between the ages of 0-5 years of age) and have MD (Mood Disorder Not Otherwise Specified, MDD or BD, with Clinical Global Impression Scale (CGI-S) score ≥ 3 and a Young Mania Rating Scale (YMRS) score ≥ 8) (N=5); 2) MD: Children who have MD without trauma (N=4); and 3) HC: Healthy Controls without trauma experience or mood symptoms (N=7). Exclusion criteria included a history of head trauma, current serious suicide risk, and co-occurring current psychosis, substance use, ASD and ID, as well as contraindications for MRI. HC were excluded if they had a psychiatric diagnosis or a first degree relative with BD, MDD, or schizophrenia. Enrollment and consent procedures for this study were approved by the institutional review board at our institution.

### Clinical and Behavioral Assessments

The following evaluations were completed by a trained child psychiatrist or child psychologist. All youth received a diagnostic assessment using the Schedule for Affective Disorders and Schizophrenia for School Aged Children—Present and Lifetime Version (K-SADS-PL) (15) to identify MD and other co-occurring psychiatric diagnoses. A supplementary module was used to assess the severity of mood dysregulation (abnormally angry or sad mood, over-reactivity to negative emotional stimuli). In addition, subjects were assessed for general psychiatric symptomatology using the Brief Psychiatric Rating Scale for Children (BPRS-C) (16), and for mood symptoms using the Young Mania Rating Scale (YMRS) (15), and Children’s Depression Rating Scale-Revised (CDRS-R) (17). Mood regulation was additionally assessed using the Children’s Emotion Management Scales: Anger and Sadness (CSMS&CAMS) (parent and child reports) (18), and Emotion Regulation Checklist (19). Subjects were assessed for trauma exposure and PTSD symptomatology using the CTQ (20), and UCLA PTSD index for DSM IV (Child and Parent report) (21). Executive function was assessed using the Behavior Rating Inventory of Executive Function (BRIEF) (22). Other clinical information obtained during the psychiatric clinical assessment included: demographic characteristics and socioeconomic status, number of medications and types, the percent of individuals with a lifelong history of psychiatric hospitalization/out of home placement, family history of psychiatric illness and substance use disorders, MRI safety screening questionnaire, and head circumference, height, and weight of subjects.

### Image Data Acquisition

Imaging data were acquired using a 3 Tesla Philips Achieva whole-body MR system (Philips Healthcare, Best, The Netherlands) with an eight-element phased-array head coil. Imaging included a T1-weighted structural MRI (MPRAGE sequence, 256×256 voxels; TR: 6.985 ms; TE: 3.15 ms; FOV: 240 mm×256 mm×180 mm; 180 slices), and a 6-min resting state acquisition (TR 2s; TE 35ms; Flip Angle 80°; image matrix 128x128; resolution; FOV 230x230mm; slice thickness 3mm; 35 axial slices).

### Data Analysis

Resting state analyses were performed using the Functional Connectivity (CONN) toolbox version 17.f (23) using routines from the Statistical Parametric Mapping software (SPM12; RRID : SCR_007037, Wellcome Trust Centre for Neuroimaging, London, UK) using MATLAB 2016b. Image preprocessing included: realignment to correct for motion, slice timing correction, and spatial transformation to standard MNI space prior to statistical analysis. The participants with movement where scrubbing flagged more than 60 time-points (⅓ of the run) across the run were rejected. Effects of nuisance variables (global, white matter and CSF signals, and movement parameters) were included in the denoising step; finally, data was band-pass filtered to 0.008–0.09 Hz.

Temporal correlations of the resting-state BOLD signal time series were examined between the left amygdala “seed” region [anatomically derived regions of interest from the automated anatomical labeling (AAL) toolbox] and the rest of the brain (seed-to-map correlation map). Based upon our hypotheses, we focused our attention on ipsi-lateral (left hemisphere) in the frontal lobe. For the group-level statistics, we used 0.001 as the cluster-forming threshold and a FWE threshold of p=0.05 (two-tailed). We modeled between group differences (CTL vs. PTS) in these maps (controlling for mean motion, age, and gender). For the correlation of left amygdala RSFC with symptom severity, we also controlled for mean motion, age, and gender.

Statistical analyses were performed using the R Project for Statistical Computing (24). Clinical and behavioral assessments were compared among the groups using ANOVA for continuous measures with post-hoc paired comparisons based on the Tukey Honestly Significant Difference method for constructing confidence intervals for the observed mean difference. The presence of co-morbid diagnoses among the groups were evaluated by Chi-square goodness of fit. Demographic and physical data are compared using similar methods for continuous and categorical measures with no correction for multiplicity.

## Results

Of the 23 subjects, 21 completed the MRI protocol. Of these, 5 were excluded due to subject motion issues, leaving the final sample size of 16 (MDT; N=5, MD: N=4, HC; N=7). Tables providing the demographic characteristics of this final imaging subgroup (**Supplementary Table 1**) and their co-occurring diagnosis (**supplementary table 2**) are available as supplementary materials. Eight of the nine subjects in the two clinical groups reported taking psychotropic medications (compared to none in the HC group), and all but two reported taking three or more medications.

### Behavioral Measures

The group-wise behavioral measures are reported in **Table 1**.

**Table 1:** Behavioral Measures.

	MDT	MD	HC	
Rating Scale	Mean ± SD	Mean ± SD	Mean ± SD	Comparisons *
Clinician Administered				
**General Symptoms**				
BPRS Total	60.8 ± 8.8	47.5 ± 15.4	24.1 ± 2.5	MDT & MD > HC
BPRS Behavioral	15.8 ± 3.9	8.8 ± 7.2	3.4 ± 0.5	MDT > HC
**Mood Symptoms**				
BPRS Mania	15.8 ± 4.0	10.8 ± 6.9	3.4 ± 1.1	MDT & MD > HC
BPRS Depression	9.4 ± 5.3	8.8 ± 5.9	3.7 ± 1.0	
YMRS	19.8 ± 5.5	9.5 ± 5.8	0.1 ± 0.4	MDT > MD > HC
CDRS	32.6 ± 11.3	24.8 ± 3.8	18.0 ± 2.2	MDT > HC
**Mood Regulation**				
ERC Lability/Negativity	43.8 ± 5.6	36.5 ± 11.6	20.0 ± 3.1	MDT & MD > HC
ERC Emotion Regulation	23.4 ± 3.6	21.5 ± 3.1	30.9 ± 1.5	MDT & MD > HC
CEMS Sadness Parent	19.8 ± 2.0	23.5 ± 3.7	22.8 ± 3.6	
CEMS Anger Parent	16.2 ± 1.6	22.0 ± 2.2	19.6 ± 2.7	MDT < MD
CEMS Worry Parent	14.2 ± 1.9	18.5 ± 0.6	18.0 ± 2.2	MDT < MD & HC
CEMS Sadness Child	24.4 ± 2.6	19.0 ± 7.0	24.6 ± 1.4	
CEMS Anger Child	21.4 ± 1.7	21.3 ± 2.6	22.4 ± 2.0	
CEMS Worry Child	18.8 ± 3.3	17.5 ± 3.9	19.9 ± 1.3	
**Trauma and PTSD Symptoms**				
UCLA PTSD Parent	20.4 ± 20.1	0 ± 0	0 ± 0	MDT > MD & HC
UCLA PTSD Child	5.0 ± 10.6	0.3 ± 0.5	1.1 ± 3.0	
CTQ Total	32.5 ± 5.6	30.3 ± 4.1	26.7 ± 2.8	
**Executive Function**				
BRIEF Global Executive Composite (T score)	73.4 ± 11.1	74.5 ± 19.4	42.7 ± 5.5	MDT & MD > HC
BRIEF Behavioral Regulation Index (T score)	67.0 ± 13.4	70.8 ± 7.2	42.2 ± 4.4	MDT & MD > HC
BRIEF Metacognition Index (T score)	70.6 ± 12.8	74.0 ±	42.0 ± 2.8	MDT & MD > HC

* Significant pairwise differences between means adjusted for familywise error based on Tukey Honestly Significant Difference.

The BPRS measures general psychiatric symptomatology. Subjects in the MDT group scored the highest on this scale (mean: 60.8, 95% CI: 49.8, 71.7), followed by MD group (mean: 47.5, 95% CI: 23.0, 72.0) and HC (mean: 24.1, 95% CI: 21.8, 26.5). A similar pattern is seen when looking at the behavioral subscale of the BPRS (MDT=15.8 (11.0, 20.6); MD=8.8 (0, 20.2); HC=3.4 (2.9, 3.9)) and the mania subscale of the BPRS (MDT=15.8 (10.9, 20.7); MD=10.7 (0, 21.8); HC=3.4 (2.4, 4.5)). The depression subscale of the BPRS suggested no difference in report of depressive symptoms between the two clinical groups; however, both clinical groups reported significantly more depressive symptoms than HC.

### Mood Symptoms

The three groups significantly differed in report of manic symptoms as measured by YMRS. Youth in the MDT group had the highest scores (mean=19.8, 95% CI: 13.0, 26.6), followed by youth in the MD group (mean=9.5, 0.27, 18.7) and HC (mean=0.1, 0, 0.5). There was no difference in report of depressive symptoms as measured by the CDRS between the two clinical groups; however, the MDT group reported significantly more depressive symptoms than HC (mean difference=14.6 (95% CI: 4.3, 24.9), p=0.007).

### Emotional Regulation

Parents reported on their children’s ability to regulate emotions using the ERC. Subjects in the two clinical groups scored significantly higher on ERC subscale 1 assessing lability/negativity (higher scores suggest greater dysregulation) and significantly lower on ERC subscale 2 assessing emotional regulation, compared to HC (ERC 1: mean difference (MDT-HC)=23.8 (95% CI: 13.4, 34.1), p= 0.0001; mean difference (MD-HC)=16.5 (95% CI: 5.4, 27.6), p=0.004; ERC2: mean difference (MDT-HC)= -7.5 (95% CI: -11.6, -3.3), p= 0.0011; mean difference (MD-HC) = -9.4 (95% CI: -13.8, -4.9), p=0.0003).

Using the CEMS—parent version, and the CEMS—child version, parents and youth were asked to rate the children’s responses to emotions of sadness, anger, and worry. On the CEMS anger subscale, parents reported that MDT youth had significantly lower coping skills in regards to anger compared to MD youth (mean difference = -5.8 (95% CI: -9.8, -1.8), p=0.006) and slightly lower than HC (mean difference= -3.4 (95% CI: -7.2, 0.4), p=0.08). Parents of MDT youth also reported that their child has significantly lower coping skills in regards to worry compared to MD (mean difference = -4.3 (95% CI: -7.5, -1.1), p=0.01) and HC (mean difference = -3.8 (95% CI: -7.0, -0.6), p=0.021).

There were no significant differences between the three groups in terms of total scores on the child ratings of anger, worry, or sadness, in the ability to inhibit sadness, worry, and anger, or in the ability to regulate the feeling of sadness and worry.

### Trauma and PTSD Symptoms

CTQ total score was on average 5.8 points higher in the MDT group than for HC, a difference which is modestly significant when correcting for multiple comparisons (95% CI: -0.8, 12.4; p=0.09). There were no significant differences in the UCLA PTSD child version amongst the three groups. In the UCLA PTSD parent version MDT youth were reported to have higher PTSD symptoms compared to MD youth (mean difference = 20.4 (95% CI: 0.7, 40.1), p=0.04), and HC (mean difference=20.4 (95% CI: 3.2, 37.6), p=0.02). The MD and HC groups both reported no PTSD symptoms on this scale.

### Executive Function

Parents of youth in both clinical groups rated their children significantly higher on all categories of the BRIEF relative to parents of HC (see **Table 1**; BRIEF Global Executive Composite T-score: mean difference (MDT-HC)=28.6 (95% CI: 13.1, 44.1), p=0.0009; mean difference (MD-HC) = 32.0 (95% CI: 15.5, 48.5), p=0.0006), with the other composite scales having a similar profile).

## Imaging Results

**Figure 1:**
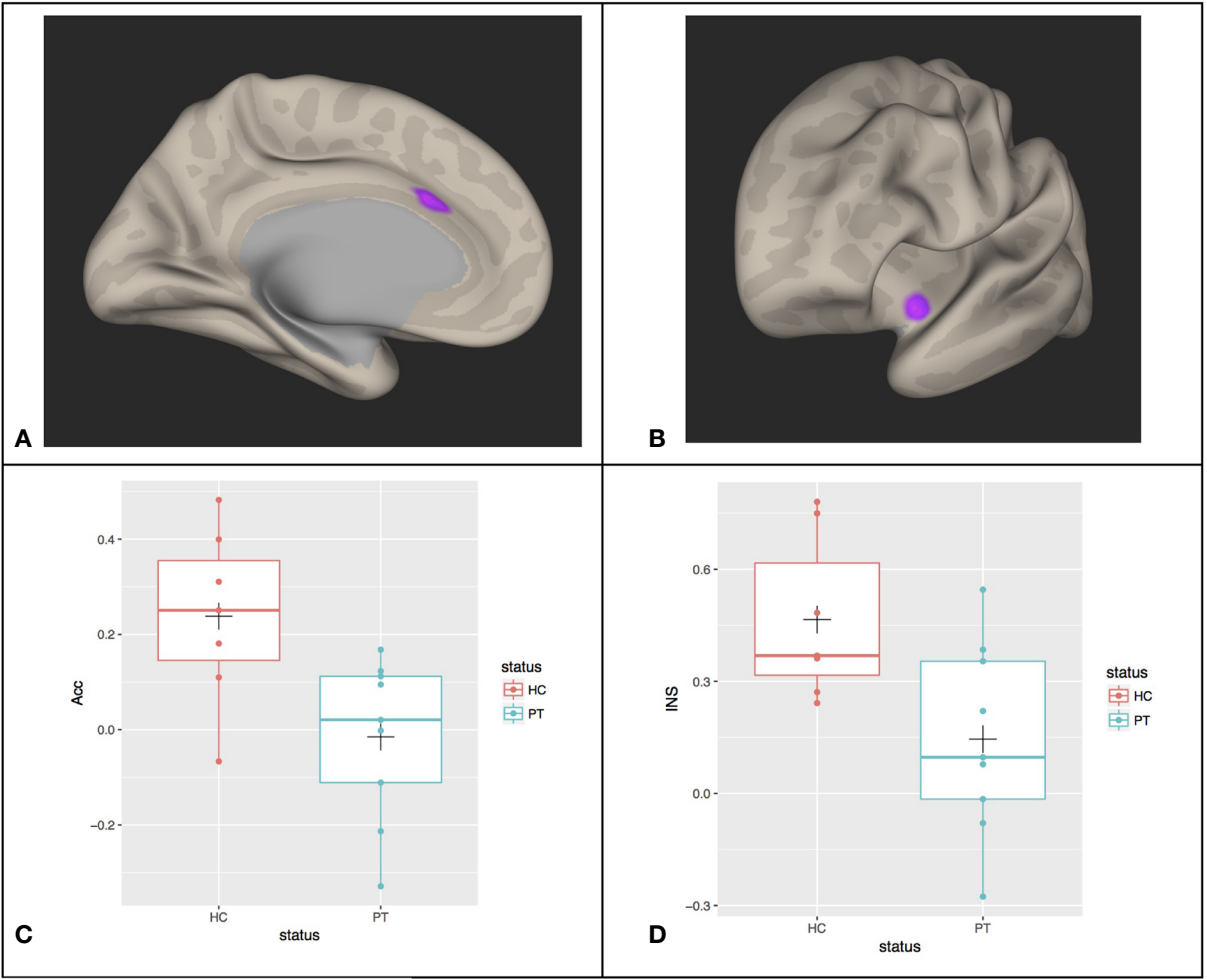
Between group differences (PT, patients; HC, healthy controls) in the functional connectivity of all voxel time courses with the mean Left Amygdala time courses in **(A)** left anterior cingulate cortex (Acc) and **(B)** left insular cortex (INS). The mean connectivity values in these regions of significance were extracted for each participant and shown in **(C, D)** with boxplots overlayed. The cross (“+”) indicates the location of the group mean.

**Figure 1** shows the between group (Patients versus Controls) differences in the left hemisphere of the connectivity of all voxel time courses with the mean left amygdala time course. Specifically, this is presented as an inflated hemispheric surface map of the medial surface of the left hemisphere, where regions that are significantly different between patients and controls are colored. There are two spatially contiguous regions identified, both in the frontal lobe. The first cluster, virtually completely contained within the left Acc region of the CONN atlas (coordinate (-4, +22, +28)), is comprised of 135 voxels and has a family-wise error (FWE) significance of 0.000284. The second cluster, residing mostly in left insula cortex (coordinate (-40, +04, -12)), is comprised of 67 voxels with FWE significance of 0.027330.

The individual average correlation values of voxels in these clusters with the left amygdala seed reference time course were extracted for each subject. **Figures 1C, D** show the box plot for the patient and control subjects for the left Acc and Insula regions, respectively. In the left Acc region, HC subjects have an average correlation coefficient between this cluster and the left Amygdala of approximately 0.24 (SD=0.18), whereas the patients’ correlation is reduced to approximately -0.015 (SD=0.17) (this difference is significant: T(14)= -2.9, p=0.013). For the insula cortex cluster, controls show approximately 0.47 (SD=0.22) correlation with the left amygdala, which is reduced to 0.15 (SD=0.26) in the patients (T(14)= -2.6, P=0.02).

**Figure 2:**
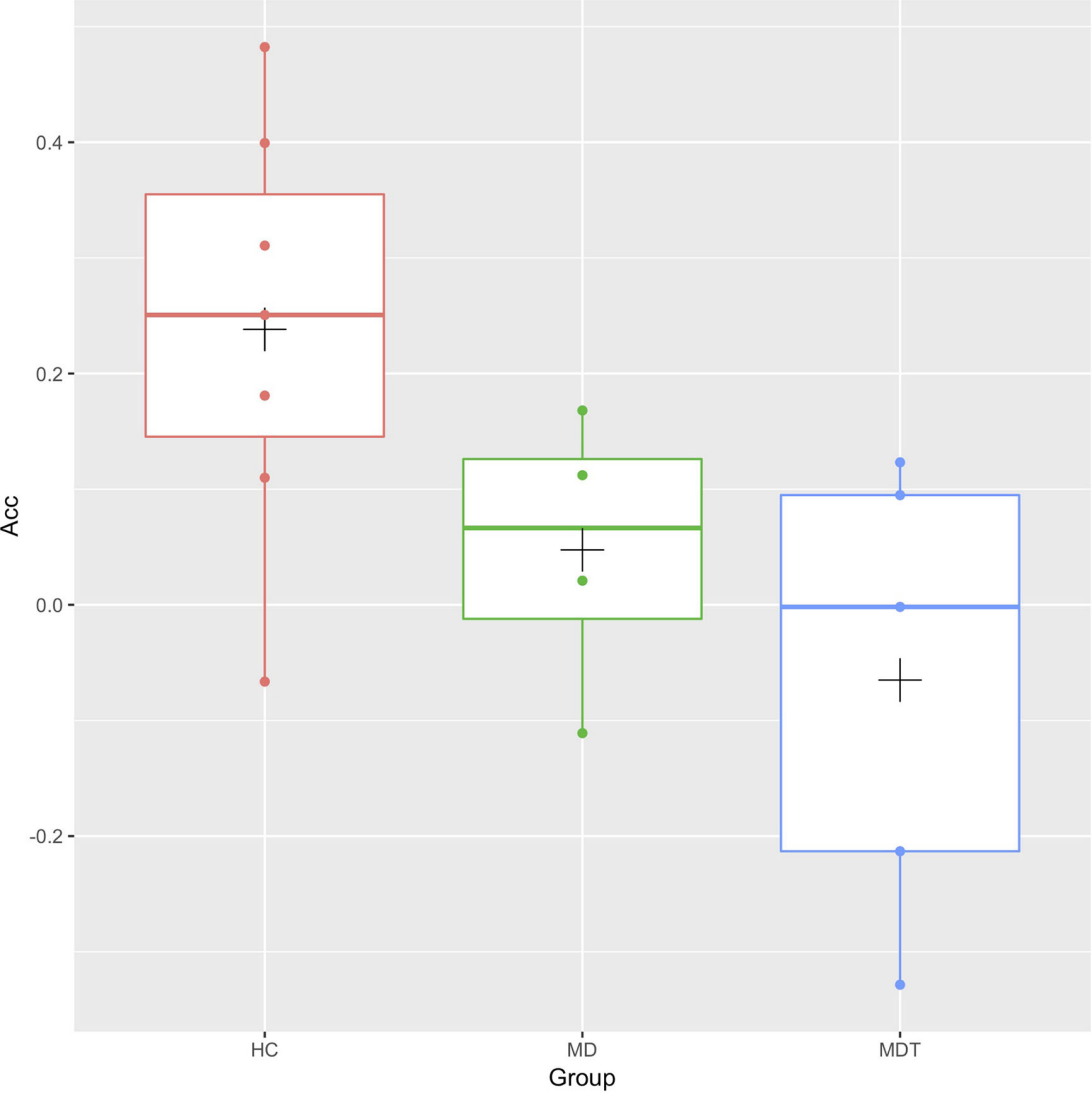
Box plots of the resting-state functional connectivity between the left Anterior Cingulate Cortex (Acc) and the left Amygdala for MDT (Mood Disorders and Trauma), MD (Mood Disorders), and HC (healthy controls) subjects. The cross (“+”) indicates the location of the group mean.

We examined if, within the patient population, there is a difference between MDT subjects and MD subjects in this resting-state correlation with left amygdala. These data are shown in **Figure 2**, with the HC subjects for comparison. No significant differences in connectivity due to trauma exposure was identified (mean difference (MDT-MD) = -0.11 (95% CI: -0.42, 0.20), p = 0.6).

### Correlation With Mood Symptoms Severity

Correlation of connectivity and CDRS in all subjects, controlling for (all subjects, age, gender, mean motion) was calculated. No significant regions were observed at the specified thresholds. We did observe a subthreshold region in the left fronto-orbital cortex (compromised of 171 voxels with a cluster forming threshold of 0.005).

**Figure 3:**
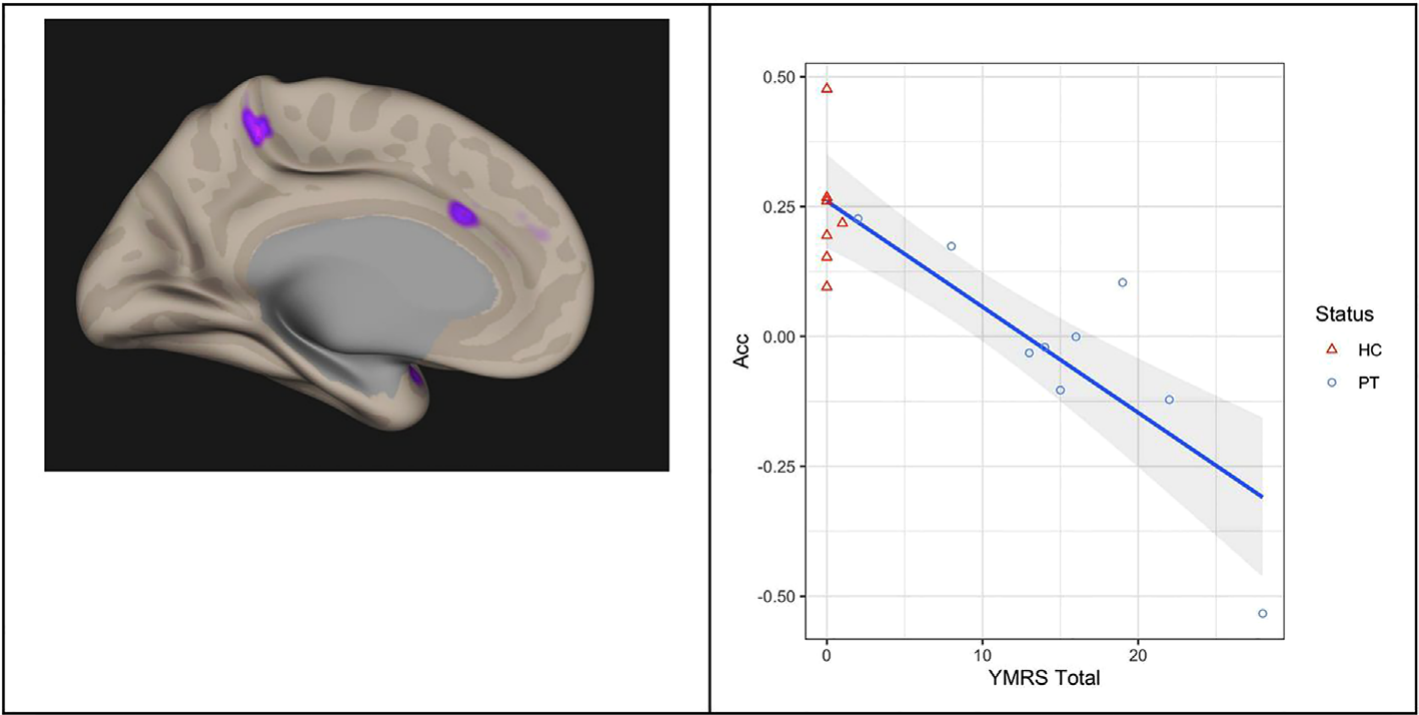
Correlation of functional connectivity and YMRS Total score in all subjects: connectivity between the left Amygdala and left Anterior Cingulate Cortex (left panel) decreases as YMRS severity increases (right panel). The line of linear best fit is shown with 95% confidence bands, with the groups distinguished by color for visualization only. Acc, anterior cingulate cortex; YMRS, young mania rating scale; PT, patients; HC, healthy controls.

Correlation of connectivity and YMRS in all subjects, controlling for (all subjects, age, gender, mean motion) is shown in **Figure 3**. Two regions are identified, but only one is in the frontal lobe. This frontal region lies within the left hemisphere Acc and paracingulate cortex. For this region, the FWE was 0.000078. Within this cluster, we plot the individual correlation value with the left amygdala seed region as a function of YMRS score. This yields a significant negative correlation (r(14) = -0.85, p = 0.0000279) of this regional connectivity with the behavioral scale: connectivity between the left amygdala and left cingulate cortex decreases as YMRS severity increases.

## Discussion

Behavioral measures suggest that MDT youth had more severe psychiatric presentations as measured by the BPRS, and more significant manic symptoms as measured by the BPRS mania subscale and the YMRS. In addition, this group had more significant difficulties regulating anger, as measured by the CEMS parent report. Interestingly, CTQ scores were only modestly elevated in the youth who have experienced trauma. This is likely because this group experienced trauma before age 5 years, and therefore did not have verbal memory of the events to report. In addition, only parents reported significant PTSD symptoms in the MDT group on the UCLA PTSD.

With RSFC study, we sought to explore the hypothesis that children with MD and trauma histories will exhibit abnormal connectivity between the amygdala and frontal lobe, when compared to children with MD without trauma and HC. Overall, while children with MD did show reduced RSFC of left Amygdala with left Acc and left Insula, we did not observe a significant difference between the subgroups of children with MD with and without trauma histories. However, when looking at both clinical groups (MT and MDT youth) together, we observe a significant correlation of RSFC of left Amygdala to left Acc, and YMRS scores. To the extent that a history of trauma may be related to increased severity of MD, the trauma factor may be exacerbating the functional connectivity alterations between these regions.

With respect to our specific hypothesis (that children with MDT will exhibit abnormal connectivity between amygdala and frontal lobe, when compared to children with MD without trauma and HC), we did not observe a significant trauma-history-specific change in RSFC separate from the diagnosis-specific changes we observed. However, the relationship between disorder severity (which is associated with history of trauma separately) and degree of RSFC change indicated the potential for an interaction that we didn’t have the power to resolve.

Anatomic regions featured in our findings included left amygdala, left insula and left Acc. The **salience network** is a ‘circuit’ that includes these regions, consisting of the two key nodes, the **anterior insula** and the **Acc**. It is also comprised of subcortical regions including the **amygdala**, dorsomedial thalamus, hypothalamus, ventral striatum, and the substantia nigra/ventral tegmental area (25). This system is integral in the top down appraisal of novel stimuli. The **insula** is involved in processing of emotions and indicated in switching between different networks, including the default mode network and central executive network. By doing so, it is thought that the insula serves to modulate behavioral responses to salient stimuli (26). Much of the salience network is comprised of structures that are also part of the limbic system; the **amygdala**, which is part of the salience network and the limbic system, is involved in the appraisal of emotionally salient stimuli from our environment and integration of these stimuli with previously processed data (27). Our findings of connectivity alterations between amygdala and Acc and insula are similar to a number of prior studies. Specifically, our findings are in line with those of Bebko and colleagues (dysregulated behaviors correlated with lower functional connectivity between the amygdala-bilateral insula) (4) and Straub and colleagues (hypoconnectivity between amygdala and dorsolateral prefrontal cortex and anterior insula in MDD) (3), in that we see altered RSFC between the amygdala and insula, as a potential bio-marker of behavioral/emotional symptoms (and disorder severity), manifesting as decreased ability to regulate emotions.

We acknowledge a number of limitations to this study. First, the sample size (final sample size of 16 (MDT; N=5, MD: N=4, HC; N=7) is admittedly small. As such, these findings and interpretations should be considered as preliminary, pending larger replication studies which can build further upon these initial observations. Second, we took a very conservative single seed-based approach to examination of RSFC (left amygdala connectivity to left frontal regions) which focused (and also ‘limited’) the scope of types of changes we would observe. While this helps reduce the number of comparisons made, it can also bias our interpretations to specific networks.

## Conclusions

In conclusion, we believe that our pilot imaging study is unique due to the population of youth studied: those with MD and histories of childhood trauma. Although our study was focused on finding a significant trauma-history-specific change in RSFC (which we did not identify), we did find that mechanisms of dysconnectivity were associated with symptom severity across clinical groups (MDT and MD). Given that there were differences in mania and coping with anger in MDT group, these seem to be clinical markers for the additional illness burden in traumatized youth with a signal in the imaging data. Clinically, screening for mania in traumatized youth, treating anger coping skills, and screening for trauma in youth with MD appear to be important conclusions.

## Data Availability Statement

The datasets generated for this study are available on request to the corresponding author.

## Ethics Statement

The studies involving human participants were reviewed and approved by UMass Medical School Human Subjects Institutional Review Board. Written informed consent to participate in this study was provided by the participants’ legal guardian/next of kin.

## Author Contributions

YD was the primary investigator designing the study and lead author. DK lead imaging effort. SH conducted all statistical analysis of behavioral measures and imaging. DP contributed significantly to researching background materials and writing the manuscript. BD assisted with design of behavioral part of the study and conducted clinical interviews. JF was the senior mentor on the project providing guidance to design of study and analysis.

## Conflict of Interest

The authors declare that the research was conducted in the absence of any commercial or financial relationships that could be construed as a potential conflict of interest.
