# Supplementary material for: Psychiatric Symptomatology, Mood Regulation, and Resting State Functional Connectivity of the Amygdala: Preliminary Findings in Youth With Mood Disorders and Childhood Trauma
Source: Front Psychiatry. 2020 Sep 18;11:525064. doi: 10.3389/fpsyt.2020.525064 (PMC7531261; doi:10.3389/fpsyt.2020.525064)
Supplement: Supplementary file 1 [file Table_1.docx]

| Supplementary Table 1. Basic demographics and physical measures. | | | | |  |  |  |  |  |  |
| --- | --- | --- | --- | --- | --- | --- | --- | --- | --- | --- |
|  |  |  |  |  |  |  |  |  |  |  |
|  |  |  |  |  |  |  |  |  |  |  |
|  | MDT | | | MD | | | HC | | |  |
|  | N | = | 5 | N | = | 4 | N | = | 7 |  |
|  | Mean | ± | SD | Mean | ± | SD | Mean | ± | SD | p value |
| **General Demographics** |  |  |  |  |  |  |  |  |  |  |
| Age (Years) | 9.8 | ± | 1.3 | 11.5 | ± | 0.6 | 9.7 | ± | 1.7 | 0.13 |
| Sex (Female: N, %) | 1 |  | 20% | 2 |  | 50% | 4 |  | 57% | 0.42 |
| Race (Caucasian: N, %) | 3 |  | 60% | 2 |  | 67% | 4 |  | 80% | 0.75 |
| Ethnicity (Hispanic: N , %) | 0 |  | 0% | 0 |  | 0% | 3 |  | 50% | 0.10 |
| **Physical Measures** |  |  |  |  |  |  |  |  |  |  |
| Height (cm) | 137.3 | ± | 10.5 | 145.3 | ± | 8.7 | 142.8 | ± | 13.2 | 0.60 |
| Weight (lbs) | 91.9 | ± | 34.6 | 101.3 | ± | 37.1 | 98.4 | ± | 16.0 | 0.89 |
| Head Circumference (mm) | 531.3 | ± | 8.5 | 527.3 | ± | 24.2 | 539.7 | ± | 12.3 | 0.46 |
| Eurion - Eurion Distance (mm) | 143 | ± | 2 | 158 | ± | 10 | 146 | ± | 6 | 0.02 |
| Glabela - Opisthocranion Distance (mm) | 179 | ± | 5 | 178 | ± | 9 | 182 | ± | 5 | 0.67 |
| Tragion - Tragion Distance (mm) | 135 | ± | 9 | 136 | ± | 5 | 134 | ± | 6 | 0.96 |
