# Supplementary material for: Psychiatric Symptomatology, Mood Regulation, and Resting State Functional Connectivity of the Amygdala: Preliminary Findings in Youth With Mood Disorders and Childhood Trauma
Source: Front Psychiatry. 2020 Sep 18;11:525064. doi: 10.3389/fpsyt.2020.525064 (PMC7531261; doi:10.3389/fpsyt.2020.525064)
Supplement: Supplementary file 2 [file Table_2.docx]

| Supplementary Table 2. Diagnoses among the groups (missing values are considered negative for the diagnosis) | | | | | |
| --- | --- | --- | --- | --- | --- |
|  |  |  |  |  |  |
| **Diagnoses (n, %)** | **MDT** | **MD** | **HC** | **p value *** |  |
| **Major Depressive Disorder** | 2 (40.0%) | 3 (75.0%) | 0 (0.0%) | 0.031 |  |
| **Severe Mood Dysregulation** | 3 (60.0%) | 1 (25.0%) | 0 (0.0%) | 0.061 |  |
| **Bipolar Disorder** |  |  |  |  |  |
| Any Type | 0 (0.0%) | 1 (25.0%) | 0 (0.0%) | 0.202 |  |
| Bipolar I | 0 (0.0%) | 0 (0.0%) | 0 (0.0%) | 1.000 |  |
| Bipolar II | 0 (0.0%) | 0 (0.0%) | 0 (0.0%) | 1.000 |  |
| Bipolar NOS | 0 (0.0%) | 1 (25.0%) | 0 (0.0%) | 0.202 |  |
| **Post-Traumatic Stress Disorder** | 0 (0.0%) | 0 (0.0%) | 0 (0.0%) | 1.000 |  |
| **Externalizing** |  |  |  |  |  |
| Any Type | 4 (80.0%) | 2 (50.0%) | 0 (0.0%) | 0.016 |  |
| Attention Deficit Disorder | 4 (80.0%) | 2 (50.0%) | 0 (0.0%) | 0.016 |  |
| Conduct Disorder | 0 (0.0%) | 0 (0.0%) | 0 (0.0%) | 1.000 |  |
| Oppositional Defiant Disorder | 2 (40.0%) | 2 (50.0%) | 0 (0.0%) | 0.118 |  |
| **Internalizing** |  |  |  |  |  |
| Any Type | 2 (40.0%) | 0 (0.0%) | 0 (0.0%) | 0.081 |  |
| Panic Disorder | 0 (0.0%) | 0 (0.0%) | 0 (0.0%) | 1.000 |  |
| Separation Anxiety | 1 (20.0%) | 0 (0.0%) | 0 (0.0%) | 0.309 |  |
| Avoidant Disorder of Childhood | 0 (0.0%) | 0 (0.0%) | 0 (0.0%) | 1.000 |  |
| Simple Phobia | 0 (0.0%) | 0 (0.0%) | 0 (0.0%) | 1.000 |  |
| Social Phobia | 0 (0.0%) | 0 (0.0%) | 0 (0.0%) | 1.000 |  |
| Agoraphobia | 0 (0.0%) | 0 (0.0%) | 0 (0.0%) | 1.000 |  |
| Overanxious Disorder | 1 (20.0%) | 0 (0.0%) | 0 (0.0%) | 0.309 |  |
| Generalized Anxiety Disorder | 2 (40.0%) | 0 (0.0%) | 0 (0.0%) | 0.081 |  |
| Obsessive Compulsive Disorder | 0 (0.0%) | 0 (0.0%) | 0 (0.0%) | 1.000 |  |
|  |  |  |  |  |  |
| * Based on Pearson's Chi-squared test |  |  |  |  |  |
|  |  |  |  |  |  |
